# A Multi-Agent System for Service Provisioning in an Internet-of-Things Smart Space Based on User Preferences

**DOI:** 10.3390/s24061764

**Published:** 2024-03-08

**Authors:** Katarina Mandaric, Ana Keselj Dilberovic, Gordan Jezic

**Affiliations:** 1 Faculty of Electrical Engineering and Computing, University of Zagreb, 10000 Zagreb, Croatia; gordan.jezic@fer.hr; 2 Department of Electrical Engineering and Computing, University of Dubrovnik, 20000 Dubrovnik, Croatia; ana.keselj@unidu.hr

**Keywords:** ambient intelligence, context awareness, Cognitive Internet of Things, Internet of Things, multi-agent system, intelligent agents, user-centric system, user preferences, negotiation

## Abstract

The integration of the Internet of Things (IoT) and artificial intelligence (AI) is critical to the advancement of ambient intelligence (AmI), as it enables systems to understand contextual information and react accordingly. While many solutions focus on user-centric services that provide enhanced comfort and support, few expand on scenarios in which multiple users are present simultaneously, leaving a significant gap in service provisioning. To address this problem, this paper presents a multi-agent system in which software agents, aware of context, advocate for their users’ preferences and negotiate service settings to achieve solutions that satisfy everyone, taking into account users’ flexibility. The proposed negotiation algorithm is illustrated through a smart lighting use case, and the results are analyzed in terms of the concrete preferences defined by the user and the selected settings resulting from the negotiation in regard to user flexibility.

## 1. Introduction

The term “smart” or “intelligent” when referring to a home, building, space, or environment is often used interchangeably with ambient intelligence (AmI), a concept that has been explored for more than two decades and was first mentioned in the late 1990s [1]. The size of the global ambient intelligence market speaks for itself, as it is estimated to be worth USD 21.61 billion in 2023, while the forecasted revenue for 2030 is USD 99.43 billion [2]. Ducatel et al. [3] emphasize the focus of ambient intelligence on user-friendliness and empowerment, while Dunne et al. (2021) highlight the role of artificial intelligence (AI) and the Internet of Things (IoT) in the realization of ambient intelligence [4]. The IoT, with its connected sensors and actuators, is essential to achieve context awareness, a key aspect of AmI. Adding cognitive capabilities to IoT devices is the focus of the Cognitive Internet of Things (CIoT) paradigm presented by Wu et al. [5]. The development of the CIoT incorporates numerous techniques from various fields, such as machine learning, artificial intelligence, context-aware computing, cybernetic–physical systems, pattern recognition, and speech recognition [6,7]. AmI and CIoT are intertwined, with overlapping application scenarios [8,9,10,11]. Although the distinction between the terms may be subtle, Jamnal and Liu [12] argue that CIoT extends the capabilities of AmI in smart environments, going beyond monitoring and supporting people’s tasks to proactively influencing users’ plans and intentions.

Numerous scientific works have explored smart homes, emphasizing the importance of recognizing and accommodating the needs and preferences of users. However, practical implementation still encounters several challenges, especially in multi-user scenarios. While the optimization of comfort for the individual user has received notable attention, the management of devices and services for multiple users simultaneously is still largely unexplored. Augusto et al. [13] point out the difficulty of satisfying a single user, let alone multiple users in the same environment. This paper addresses the challenge of managing and satisfying individual user preferences in multi-user scenarios in which all users are inevitably affected by service device settings. For example, consider the case of managing the ambient conditions in a room based on the preferences of multiple users, in which case it is unlikely that all users have the same preferences.

The research results presented in this paper show that our multi-agent system effectively addresses multi-user scenarios by accommodating diverse preferences and resolving conflicting requirements, benefiting all individuals involved. Multi-agent systems are a valuable solution for complex systems in which the division of tasks makes a big difference in effectiveness. Moreover, context-aware systems require intelligence and autonomy, which are attributes of software agents [14]. In this context, our system employs specialized agents that represent users and services. Agents representing users, predict the preferences for an unseen context based on detected and established user preferences via artificial neural networks, and they represent users in the negotiation process with other user agents. In this paper, a novel negotiation process that utilizes agent grouping based on flexibility factors in conjunction with user preferences is proposed. It also enables effective negotiation in which user agents are equal without neglecting any user. At the same time, criteria are maintained that do not allow any user agent to obstruct the negotiation process by forcing the decision to favor their own preferences over those of other agents. To ensure interoperability between different devices of the same type, since not all devices have the same ranges, options, etc., we introduce a space agent tasked with aligning preferences with the devices’ capabilities. We chose to focus on lighting not only because it is obviously one of the ambient conditions that are the sources of arguments in common areas at home and at work but also because it has a great impact on humans, as pointed out by Tomassoni et al. [15], who emphasize the influence of light intensity and color on people’s emotions and psyches. A statement by Chew et al. [16] underlines the complexity and importance of smart lighting: “*it can be concluded that the future of smart lighting development is a multi-disciplinary research area; smart lighting has the potential to provide the platform to bring advancements in key research areas pertaining to energy efficient buildings, human health, photobiology, telecommunications and human physiology to our living rooms and offices*”. Another service that is prone to similar problems in a multi-user setting, i.e., conflicts between users with different preferences, is smart heating and cooling.

In summary, the key contributions presented in this work are as follows:

- The definition of a service provisioning solution for multi-user scenarios in an Internet-of-Things smart space based on the optimization of service parameters, depending on the context, available devices, users, and users’ preferences.
- The solution architecture equipped with intelligent software agents representing users and spaces for smart space service provisioning.
- A negotiation organization and negotiation process for software agents in the dynamic adaptation of ambient conditions based on context, presenting users’ preferences and flexibility factors.

In addition to these main contributions, the challenges associated with the simultaneous provision of services to users with different desires and needs are also discussed, highlighting the lack of solutions for this type of scenario. Furthermore, the intricacies of defining preferences are explored, and the extent to which contextual factors are considered in this process is examined.

The paper is organized as follows. Section 2 presents the related work. Section 3 describes the methodology behind the development of the proposed system and the negotiation algorithm. Section 4 describes the selected use case and displays the results of the negotiation algorithm in the conducted experiments for the selected use case. The paper ends with Section 5, in which a discussion and conclusions concerning the results of this research, as well as plans and ideas for future work, are presented.

## 2. Related Work

Smart homes are not new, but they have gone through a significant evolution to reach their present state. In the beginning, the burden of automation fell on users, resulting in homes that were programmable but not yet aligned with the modern understanding and expectations of smart homes. As a case in point, consider that, in 1991, Stauffer [17] described a smart house as an ”*enabling system that provides the common resources needed for home automation*”. For many users, this was just an added pressure element and obligation because, for example, they would forget to turn on some of the various modes of the system. As an illustration, consider a “not at home” mode that would turn off all the lights, lock the door, turn off the heating, turn off the security alarm, etc. Also, initially, the settings of such systems were quite complicated and unintuitive. It is obvious that, to achieve truly smart homes, they must be adapted to the user and not the other way around, when the “brains” of the smart environment are smart users [18]. The system must listen to its users and adapt to their needs and desires by programming itself; users must not spend more time adjusting the system than it would take to perform an action on their own.

It is evident that contextual awareness, a prerequisite for AmI, is significantly important. Contextual awareness is the foundation without which it is impossible to start talking about ambient intelligence or CIoT and, therefore, also about providing ambient services to the user. Lovrek [19] points out that context awareness had become a subject of research thirty years ago and noted its promising and challenging impact on the functionality, efficiency, and complexity of systems. It is also recognized in the field of the Internet of Things by Perera et al. [20], which further confirms the connection between IoT, ambient intelligence, and context awareness. According to Dunne et al. [4] the research community agrees that context awareness is critical in the development of ambient intelligence systems, as the awareness of context enables the accomplishment of complex tasks through an understanding of the states of the user and the environment, allowing for greater precision in the predictability, assistance, and handling of multiple users in the same space. In the system proposed in this paper, the preferences for a selected service are defined by users based on the context recognized in a smart environment. This fundamental aspect of the solution proposed in this paper ensures adaptability and emphasizes the importance of context awareness in navigating changing ambient conditions.

The idea of smart homes enriched with cognitive technologies has long been discussed, as they take the cognitive burden off users. This can be seen, for example, in a large amount of research, much of which focuses on energy savings with the help of machine learning and artificial intelligence [21,22]. User comfort is put at the center of the focus of numerous works, alongside other factors, such as energy saving. Rasheed et al. [23] use mathematical optimization models to minimize power consumption while taking into consideration user comfort, which is modeled based on user preferences, weather conditions, and appliance classes. Different comfort constraints apply to different appliances; for example, air conditioner usage is essential in the summer to ensure user comfort, but it leads to higher electricity bills.

Artificial neural networks (ANNs) have established their place in the development of smart environments since they are used for automation and the control of appliances, activity recognition, classification, etc., with various types of learning used for ANNs. In 1998, Mozer [24] used a feed-forward neural network with reinforcement learning to develop ACHE, a smart home system that aims to predict user action in order to save energy and satisfy users. A deep attentive tabular neural network (TabNet) was used by Ani et al. [25] in 2023 for prediction and control tasks through imitation learning to automate a control, and they pointed out that it is more suitable for the task than reinforcement learning. Health and well-being monitoring has been improved and raised to a different level with the introduction of IoT, as is evident in a large number of papers published on the subject [26,27,28]. It has proven to be very helpful in providing older people with the opportunity to stay independent and relieving burdened health systems. These application ideas and concepts have received more attention, given the COVID-19 pandemic [29,30]. Despite the positive aspects, there is resistance towards some forms used in ambient assisted living (AAL) system proposals, such as cameras and microphones, as was found in a survey carried out by Igarashi et al. [31].

Various approaches to the development of smart environments have been explored, one of which is the integration of software agents. Among their three primary attributes—autonomy, the ability to learn, and cooperativeness [32]—lies the foundation for their further growth and development. These attributes are the key to developing additional abilities such as reactivity, proactivity, coordination, negotiability, and sociability that contribute to the development of personalized user experiences in smart homes. They have been integrated into smart environments in many various ways, for example, in energy management [33] and home automation. Park et al. [34] describe a reinforcement learning-based system for lighting that balances two factors, human comfort and energy savings, with the goal of achieving energy savings without neglecting and sacrificing user comfort. Their system learns users’ behavior patterns and the environment, but it is focused on one user. Belief–desire–intention models are often used in the design of software agents, like in the work by Sun et al. [35]. Their multi-agent system is described in an illumination control use case. The system does not differentiate between different users, but the user inputs require illumination control through the user interface. The BDI model guides the decision-making process in order to achieve energy savings and set the illumination conditions to complement the current activity of users. In their study, Amadeo et al. [36] introduce the COGITO platform for cognitive buildings, focusing on energy efficiency, security, and user comfort. Within their cognitive office framework, they describe four agents responsible for position detection, activity recognition, curtain management, and lighting management. The environmental settings are adjusted according to the detected activity, which includes possible states of no activity, meetings, desk work, or free time.

Undoubtedly, the research and development of smart spaces have come a long way, but they continue to progress since there is much to be yet covered. While there are many advanced solutions with respectable and significant results, works focus predominantly on single-user scenarios in smart environments, as pointed out by Oguego et al. [37]. This highlights the complexity of multi-user environments. Consequently, there is only a limited number of solutions that address this significant challenge. This is particularly evident in human activity recognition, which requires more attention since accurately detecting the actions of multiple individuals in shared spaces can unlock new application possibilities [38]. Cook [39] observes that only a limited number of researchers have endeavored to address the complexities arising from multiple users within the same smart environment. Even though this observation was made in 2009, the scenario with multiple users is still a problem and a challenge, especially when taking into consideration their preferences.

However, there are works that deal with this challenging issue in different forms, focusing on different AmI scenarios not necessarily in the smart home. Valero et al. [40] present their MAS platform, Magentix2, which improves the adaptability and efficiency of smart environments by defining user profiles, controlling access to services, and optimizing the information flow for inhabitants by tracing their behavior. Their platform provides an effective solution to the challenge of controlling access to smart home functions and devices through the definition of organizational roles and organizational units in living spaces. However, it does not address the issue that arises when two agents, carrying the same weight, i.e., role, may have different preferences. If users have the same role, conflicts may arise when multiple users attempt to set device settings according to their individual preferences.

Negotiations can be mediated or non-mediated, like the non-mediated bilateral multi-issue negotiation model proposed by Sanchez-Anguix et al. [41], which is illustrated through the example of a product fair with separate agents representing buyers and sellers. These agents incorporate the negotiation attributes of their client, i.e., buyer or seller, and then search for and attract potential deals, aiming to minimize the negotiation process and save valuable time. An approach with a home agent (mediator) for the negotiation process between user agents and device agents is proposed by Loseto et al. [42]. The goal is to optimally fulfill the user agent’s function requests. While it is based on user needs and preferences for service functions, conflicting situations in which users have different preferences for the same functionality are not covered. Muñoz et al. [43] use argumentation in their proposal for a television service that acts as a mediator, taking into account the preferences of users and television programs to make recommendations aimed at satisfying all users present. Argumentation is also used by Oguego et al. [44] to manage conflict situations, focusing not on conflicts between multiple user preferences but, rather, on reconciling user preferences with device settings, especially with regard to safety concerns.

AmI systems, particularly AAL systems, are expected to adjust and customize their services in order to achieve maximum user comfort. This goal becomes unattainable if the system falls short in gathering information and generating knowledge about the user and comprehending their preferences [45]. Preferences are not always clear-cut and easily presented; for instance, consider lighting. While it is easy to define a preference when the options are limited to “on” and “off”, things get more complicated when introducing lighting fixtures that offer multiple colors and intensity levels. With more devices and options, there are also more ways to combine preferences, especially when more context is included in the definition of preferences. The domain of context recognition presents a complex challenge, providing numerous research possibilities, considering the complexity of obtaining and defining context attributes despite context awareness having been a research topic for more than three decades. Moulouel et al. [46] address the challenge of context abnormalities within partially observable uncertain AAL environments with an ontology-based framework. Their approach integrates a probabilistic context reasoning formalization, aiming to effectively address the incompleteness of knowledge in such environments.

While it is important to acknowledge the users’ concerns about their data security and privacy, this paper will not focus on those issues. This paper will focus on the negotiation process in a multi-agent system in which agents represent users’ preferences with the aim of achieving a decision that suits all users.

## 3. Methodology behind a Multi-Agent System for Service Provisioning

### 3.1. Agent Types, Organization, and Architecture

The envisioned multi-agent system was designed with the goal of achieving users’ satisfaction at its core, representing a user-centric approach. But this goes beyond one user; the focus is on all present users, each with their own unique preferences that can change at any time. Therefore, it naturally follows that each user is assigned a dedicated agent responsible for advocating for their preferences in the negotiation process. A more comprehensive explanation of preferences, including how user agents observe and learn users’ preferences and subsequently manage them, is presented in Section 3.2.3. The number of user agents in the negotiation process equals the number of users present in the specific smart environment.

During the design process of the negotiation algorithm, several options were considered to reach agreements between agents representing users and reduce their preferences to a single selection. Conitzer [47] discusses numerous decision rules with various complexity levels, ranging from the simple and straightforward plurality rule to the more complex Kemeny rule. The plurality rule selects the most preferred alternative, and complementarily, the antiplurality rule selects the least disliked choice. Furthermore, he describes more complex approaches such as the Borda rule, which utilizes a ranking system and integrates a scoring system based on each negotiator’s preference for option placement—whether it is first or last. In Kemeny’s rule, which has been previously tested for preference aggregation [48], alternative rankings are aggregated, ultimately proposing one that minimizes the distance from the entered rankings.

The goal of our proposed solution is to create a decentralized system without a centralized “brain” that makes a decision after receiving the preferences of all users present. This opened several questions that had to be carefully approached, one of which was the course of negotiations. For example, when “chain” negotiation is taken into account, the first agent submits their preference to the second, the second proposes a joint decision that they agree with the first agent and then sends it to the third agent, who, with the second agent, checks the joint decision and sends it on to the fourth, who does the same, etc. From the first to the last agent, i.e., after one round, the proposal for a decision is possibly very different from the preference of the first agent. Thus, a certain course must be set in place that does not “forget” or neglect any participant, no matter their position in the negotiation process.

As expected, the negotiation process becomes increasingly complex when users exhibit a wider range of diverse preferences. Also, restarting a negotiation process from the start every time a user enters or exits the smart environment would be inefficient. In both of these scenarios, another independent entity should handle these problems. This will be handled by the smart space agent. For the first scenario, when user agents cannot reach an agreement in the allowed number of negotiation rounds, the smart space agent collects all of their preferences and calculates a setting that will attempt to satisfy all the users to the maximum. This centralized solution is only resorted to when no agreement has been reached in the permitted number of negotiation rounds, which is defined according to the number of users and the scope of the negotiation; i.e., it is not the same when they are negotiating one setting as when they are negotiating more settings. In the second scenario, the smart space agent will check with the user agent that just registered whether the current setting satisfies them and try to reach an agreement without involving all other agents and triggering a new negotiation process. As expected, this approach is not always successful, depending on the difference between the current setting and the preference of the new user, so the negotiation process is restarted.

Another role of the smart space agent is to act as a middleware element. For instance, when considering lighting preferences, it is important to note that not all lighting fixtures in different smart environments share identical settings. These include variations in maximum intensity values and color options. Furthermore, the smart space agent monitors the ongoing context and updates the user agents. This eliminates the need for user agents to gather sensor readings from multiple sensors on their own, which means less traffic on the network and enables interoperability because the smart space agent passes sensor readings to the user agents in an agreed notation understood by all agents. This means that the smart space agent is like a specialist dedicated to the specific smart environment. It can be considered in future works that one smart space agent is in control of more spaces, like a whole house or building floor.

The described multi-agent system (MAS) has two agent types per smart environment: user agents *u_agent_n_*, and a smart space agent *s_agent* marked as follows:

(1) $$\mathrm{MAS}=(s\_agent,u\_agent_{1},u\_agent_{2},\ldots ,u\_agent_{i},\ldots ,u\_agent_{n})$$

where the number of user agents, *n*, equals the number of users present in the smart environment; i.e., each user agent *i*, *u_agent_i_*, is associated with one user.

**Figure 1:**
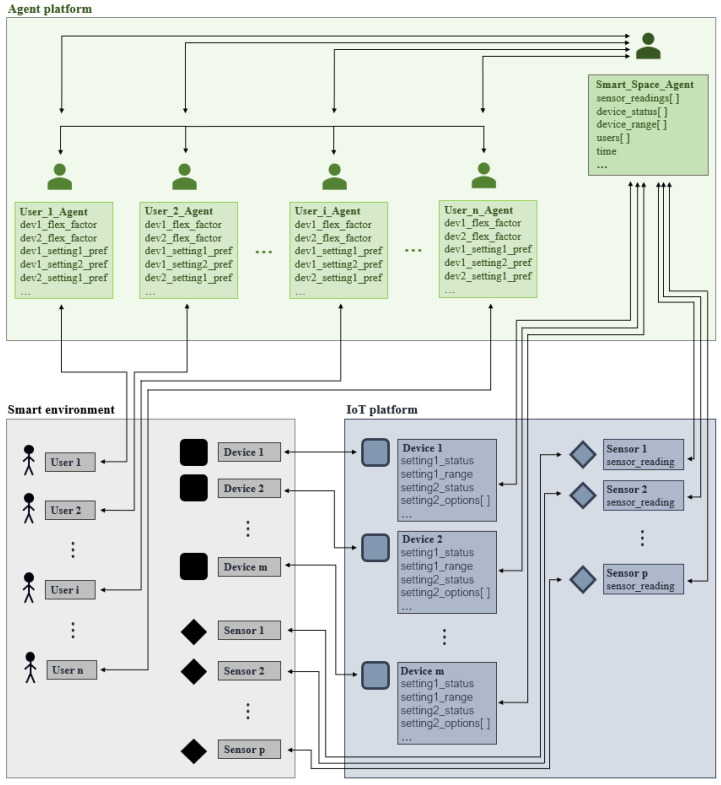
System architecture.

The proposed system architecture is shown in Figure 1. It illustrates the relationship between users, agents, devices, and sensors placed in three different environments—the physical environment, the IoT platform, and the agent platform.

The physical environment, i.e., the smart environment, is equipped with devices and sensors, the selection of which depends on the selected service to be provided to the currently present users in the environment. The sensors are needed to assess the state of the environment, i.e., to achieve context awareness so that the devices can be controlled based on their possible settings and options to manipulate the ambient conditions to match the users’ preferences.

As explained earlier, it is evident that the user agents do not access devices and sensors on their own. The responsibility for monitoring device states and sensor readings, as well as forwarding and processing them for the user agents, lies with the smart space agent, which connects to them via an IoT platform.

The IoT platform hosts virtual representations of devices and sensors within the smart space. The current status of each setting of a device (*setting_status*), the possible range (*setting_range*) and/or options (*setting_options[]*), as well as the sensor readings (*sensor_reading*) are stored there.

An agent platform hosts two types of agents: the smart space agent and user agents. The number of agents varies based on the number of present users, as explained previously. The smart space agent gathers information from the IoT platform about devices and sensors, as well as information about present users, the current time, etc. The obligation of the smart space agent is to provide the user agents with the information relevant to them according to the implemented use case. Each user agent holds information about the user, such as their preferred settings for devices (*dev_setting_pref*) based on the selected use case, i.e., service, and their flexibility factor for the specific service, i.e., the device that provides the specific service (*dev_flex_factor*). The prediction of preferences and the role of flexibility factors are described in detail in Section 3.2.3 and Section 3.3.

Also, users do not access the IoT platform on their own, as it is intended to be a truly intelligent service provision. When users want to make any changes to the environment, they do so through their user agent, for example, to enter new preferences and modify or delete existing ones (*dev_setting_pref*), as well as to redefine their flexibility factor for a certain device (*dev_flex_factor*).

In Section 3.5, an in-depth explanation of the behavior of each agent type in the negotiation process is provided. An overview of the implementation of the proposed system for a selected use case, including an analysis of the results of the tested scenarios, can be found in Section 4.

### 3.2. Context

It can be said that the effectiveness and precision of the proposed multi-agent system is proportional to the collected context. A richer context enables a more detailed definition of user preferences, which is causally connected with the ability to satisfy user preferences to the maximum. But it should be emphasized that a richer context affects the level of complexity and, in certain cases, causes an increased number of interactions between agents when making a decision.

The relevant context, *C*, incorporates the following three items:

(2) C=(SR,DS,P)

where the following applies:

- *SR*—represents sensor readings that are used to sense the ambient conditions;
- *DS*—represents the device status of actuators can alter the ambient conditions;
- *P*—represents currently present user preferences.

#### 3.2.1. Sensor Readings

Relevant sensor readings can come from the same sensor type but different locations. For example, when deciding on the optimal temperature for their home, users require information regarding both the outdoor and indoor temperatures. The sensor readings are marked as follows:

(3) $$\mathrm{SR}=({\mathrm{sr}}_{1},{\mathrm{sr}}_{2},\ldots ,{\mathrm{sr}}_{j},\ldots ,{\mathrm{sr}}_{m})$$

The total number of sensor readings, *m*, indicates the number of sensor readings relevant to the service in focus. For different services, different sensor types are needed to assess the context. For example, in the case of an AC control system, temperature sensors are essential.

#### 3.2.2. Device Settings

Devices, i.e., actuators, are crucial in accommodating user preferences through the manipulation of various settings to align with users’ preferences. Device settings are marked as follows:

(4) $$\mathrm{DS}=({\mathrm{ds}}_{1},{\mathrm{ds}}_{2},\ldots ,{\mathrm{ds}}_{j},\ldots ,{\mathrm{ds}}_{m})$$

#### 3.2.3. User Preferences

User preferences can be detected or observed and then analyzed and stored using various methodologies. The user preferences part of the negotiation process at a given moment in time is marked as follows:

(5) $$P=(p_{1},p_{2},\ldots ,p_{i},\ldots ,p_{n})$$

where the total number of preferences, *n*, indicates the number of present users in a particular smart environment. Users determine their preferences based on the relevant context for each preference. For instance, when configuring an AC system, considerations may include outdoor and indoor temperatures, the time of day, the season, and ongoing activities. In this proposed system, users define their preferences solely based on sensor readings *SR* and device settings *DS*, as seen in (6). This means there is no need for them to factor in others’ presence and their preferences, as this aspect is managed during the negotiation process between agents. Device settings are important since the different smart environments and devices that are part of it do not necessarily offer the same service options; for example, consider various modes of an AC system.

(6) $$f\left(U\right):C_{SR,DS}\to P$$

These preferences can be realized either as precise, exact specifications or as a range of values corresponding to the condition under which it would accommodate the user. Users can specify a preference with an exact value *x* for a particular device setting *e* (*ds_e_*) for a particular context *C*. For example, a user may define their temperature preference to be 22 °C in their home during the day in winter and 17 °C at night. Alternatively, they could express a broader range of acceptable values, finding satisfaction as long as the value for e.g., heating—remains within a specific range or meets a certain threshold of intensity. This means that satisfaction is achieved and upheld as long as the temperature value is greater than *x*, denoted as “>*x*”. In scenarios where a single device setting is in focus within a smart environment, as seen for preference *a* (7), the preferred numerical value *x* is attributed to the device setting *e* (*ds_e_*). If, for instance, the preference encompasses multiple device settings—such as color and intensity for lighting—then the preference consists of two values, as demonstrated in preference *b* (8).

(7) $$p_{a}=\left[{\mathrm{ds}}_{e}:x\right]$$

(8) $$p_{b}=\left[\left[{\mathrm{ds}}_{e}:x\right],\left[{\mathrm{ds}}_{f}:y\right]\right]$$

### 3.3. Flexibility Factor

The maneuvering of preference modifications in the negotiation process is directed through the implementation of the flexibility factor. Through this approach, users retain the ability to articulate their precise preferences while incorporating supplementary information regarding the extent to which modifications can be applied—a notion referred to as “stretching” the preference.

The flexibility factor is modeled after the Gaussian function (9). The Gaussian function is applied in the following way: along the *x*-axis, numerical values corresponding to adjustable device settings within an environment are plotted, while the *y*-axis represents the associated user usefulness values. The peak of usefulness, i.e., the mean of the Gaussian function μ, corresponds to the user’s preferred value. As a result, the utility function consistently centers around the precise user preference value. The introduced flexibility factor essentially represents the standard deviation σ of the utility function.

(9) $$f\left(x\right)=e^{-\frac{{\left(x-\mu \right)}^{2}}{2\sigma^{2}}}$$

**Figure 2:**
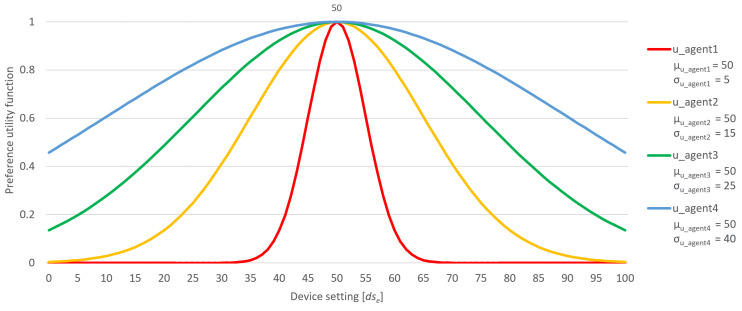
Representation of the exact same preference with different flexibility factors.

As the flexibility factor increases, the Gaussian utility function becomes wider and wider, as shown in Figure 2. In this figure, four users share an identical exact preference, denoted by the numerical value 50, which corresponds to the peak value of their utility functions. However, these users have different flexibility factors, with the most adaptable user shown in blue. This results in a slower decline in utility compared to the original user preferences, meaning that users with higher flexibility factors are more adaptable. During the negotiation process, it is easier to negotiate with them since they are more willing to accept propositions that deviate from their exact preferences.

**Figure 3:**
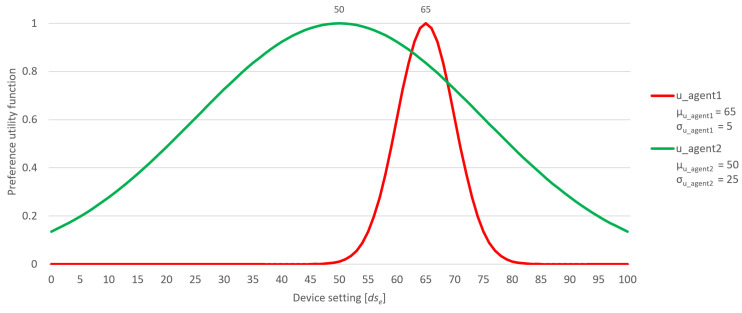
Users with different exact preferences and different flexibility factors.

Consider an Illustrative point: the wider utility function of a user with greater flexibility might encompass the narrower utility function of a less flexible user. This scenario is portrayed in Figure 3, where users, despite not sharing identical preferences, experience a smoother negotiation process due to the flexibility demonstrated by the user depicted in green. An in-depth analysis of this and other different scenarios is given in the subsequent sections of the paper. When a user sets their flexibility factor to 0, it signifies that only their precise preference is deemed acceptable, leading to an immediate decline in their utility function if the setting deviates from their exact preference. On the other hand, if a user opts for a very high flexibility factor, their utility function consistently maintains a high value for any given device setting.

As is evident, the number of preferences that need to be assigned to all the variations of the context grows exponentially, depending on the number of elements that make up the context. The more context information is available, the more precisely users can define their preferences, which makes it easier for the system to adapt the services to the users’ needs. Expecting users to enter all of these preferences manually is not realistic, and it contradicts the concept of a truly smart service, which aims to minimize user effort. Therefore, users are not required to enter all of their preferences. Instead, the system takes on the task of learning their preferences through observation. In this way, the system can predict a user’s preferences for a given context without the user having to explicitly enter anything. The task of predicting preferences is performed by the user’s agent, which is equipped with an artificial neural network (ANN) to predict preferences. Since preferences are defined based on context, the input to the ANN consists of context information, and the output is the predicted corresponding user preferences. The training of the network is based either on preferences entered by the user or on observations of the preferences selected by the user over a certain period of time, which should cover different contextual situations in order to create a reliable foundation for preference prediction.

### 3.4. Negotiation Group Organization

When designing the algorithm, the constant thought was also to minimize the number of messages exchanged by the agents. Therefore, the grouping of users was immediately considered. Grouping users, i.e., user agents, is a logical option to reduce the number of preferences that “confront” each other by connecting agents according to a certain criterion. It is intuitive to group agents according to their preferences, i.e., to group all agents who have closely related preferences into groups for which a joint decision will not require any of them to deviate more from their exact preference. Closely related, i.e., similar, preferences can be defined more strictly or more loosely. For example, in smart lighting, user groups can be defined using different colors (red, green, blue, etc.), or the colors can be divided into cool and warm colors. If the number of users with similar preferences was decisive for a decision, their reduction must definitely have an impact on the definition of a uniform decision. The number of users in groups is clearly visible here, making the scenario in which a change occurs easier to handle. Even though this is an option worth exploring, there are some drawbacks. The context is constantly changing, so when the context changes, it is necessary to reconstruct the groups, as one or more users may have changed their preferences for a new context. In addition, the described flexibility factor and the scenarios in which users enter and leave a smart environment must be taken into account. Since agents are “hidden” in groups, it is more difficult to detect and implement a change in device settings when a user with a low flexibility factor enters or leaves the room. For example, in the smart environment, there are several groups with relatively high flexibility factors and the final decision between the groups was made based on the smallest group, which contains a user with a low flexibility factor in addition to users with higher flexibility factors. If this user is eliminated, the previous decision is no longer correct. This is a simple example, but in scenarios where only a small variation in flexibility factors and the number of users present have influenced the decision, any change in presence in the smart environment requires renegotiation, as the user agents are looking to maximize their utility function and could use the change to their advantage.

**Figure 4:**
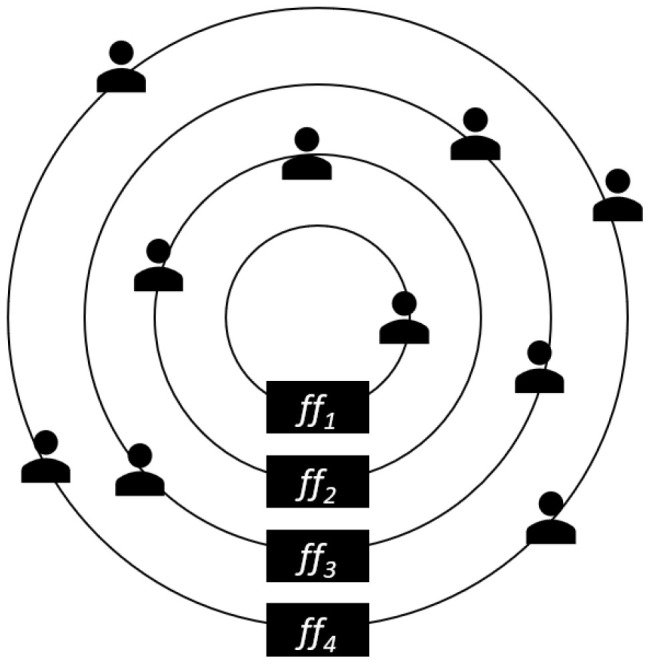
Grouping users by flexibility factor in circles.

**Figure 5:**
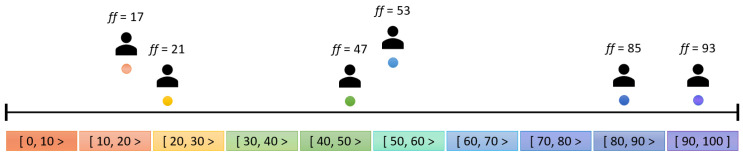
Grouping approach with fixed groups.

The second idea, which seems more complex at first, facilitates the negotiation process in many ways. The agents must be grouped according to their flexibility factors. This is better than the previous approach because the flexibility factor is constant, i.e., it only changes when the user changes it. The proposed negotiation process ensures that no user is “left behind”, and the decision is constantly subjected to review by all participants through negotiation rounds. As can be seen in Figure 4, users are placed in concentric circles according to their flexibility factors, with the inner circles accommodating the users with the lower flexibility factors. As the diameter of the circle increases, so does the flexibility factor of the users in that circle. The organization of these circles, i.e., groups, is in the hands of the smart space agent. The question arises as to how large the individual groups should be, i.e., how wide the value range of the flexibility factor is that defines a group. Initially, a fixed definition of the minimum and maximum flexibility of each group was considered, but this approach turned out not to work well in the vast majority of cases. For example, two users with a similar flexibility factor in a space could be assigned to different groups, which would constitute the mistake shown in Figure 5. Here is the most extreme example: agents representing users with flexibility factors 19 and 21 are assigned to different groups despite the slight difference in their flexibility factors, which is only 2.

**Figure 6:**
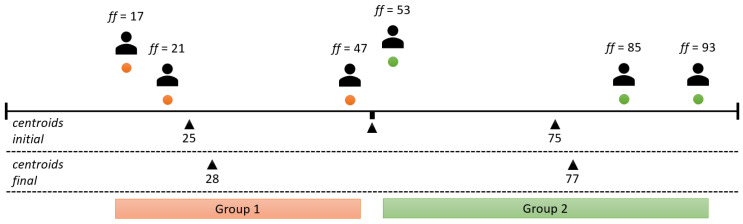
User agent grouping example using two centroids (*SC* = 62).

**Figure 7:**
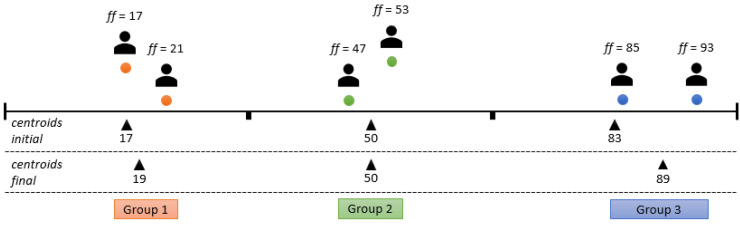
User agent grouping example using three centroids (*SC* = 91).

**Figure 8:**
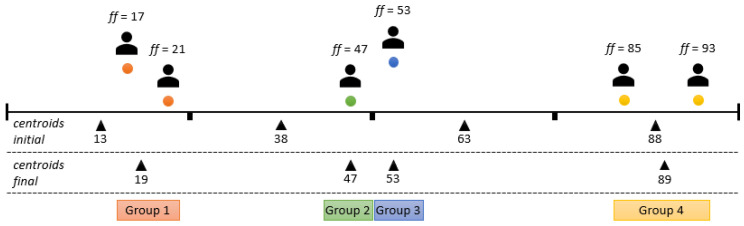
User agent grouping example using four centroids (*SC* = 94).

The solution chosen for this problem was the K-means clustering algorithm [49], an unsupervised machine learning algorithm for determining clusters—in this example, agent groups with similar flexibility factors. The mentioned algorithm fulfilled the required characteristics and performed the task at a high level, but with a drawback; i.e., it is necessary to determine the number of clusters in advance. For this reason, the smart space agent has the task of calculating the optimal grouping for different numbers of clusters using the silhouette method. The selected number of clusters is the one with the highest silhouette coefficient, which is calculated using two distances. The first distance is the distance between the data point (in this case, an agent with a flexibility factor) and the center of the cluster to which it is currently assigned. The second distance is the distance to the nearest center of the cluster that it is not assigned to [50]. For the presented example of the agents present, i.e., users with different flexibility factors, the analysis of the number of clusters is shown in Figure 6, Figure 7 and Figure 8.

The initial centroids were set at the midpoints of the range of the original division. As can be seen in Figure 6, the initial values for the two centroids were, thus, set to 25 and 75. Considering the agents present, the centroids were shifted to 28 and 77 at the end of the method with a silhouette coefficient (*SC*) of 62. The division into these two groups was obviously inappropriate, as agents with flexibility factors of 47 and 53 were separated; i.e., they were grouped with agents for which the difference in the flexibility factors of the agents from the group was above 20. In this clear example, the division into three groups is a simple visual solution, as can be seen in Figure 7. This approach outperforms the division into two groups, as indicated by a higher silhouette coefficient (*SC* = 91), which underlines the better grouping accuracy. Figure 8 shows the grouping into four categories. Despite a (slightly) higher silhouette coefficient (*SC* = 94) compared to the division into three groups, it is important to note that this division into four groups entails “overfitting”. It isolates agents with minor differences in flexibility factors (in this example, agents with factors of 47 and 53) into separate groups, even if the differences are minimal. To avoid this, the maximum number of clusters is determined by rounding up the division of the current number of user agents by two.

Based on this example, the choice is to divide the user agents into three groups. This choice ensures an optimal flow of the negotiation process, as the division into two or four groups leads to an increased exchange of messages. This is due to the fact that agents with significantly different flexibility factors are grouped together, or agents are separated despite minor differences in their factors.

### 3.5. Negotiation Process

**Figure 9:**
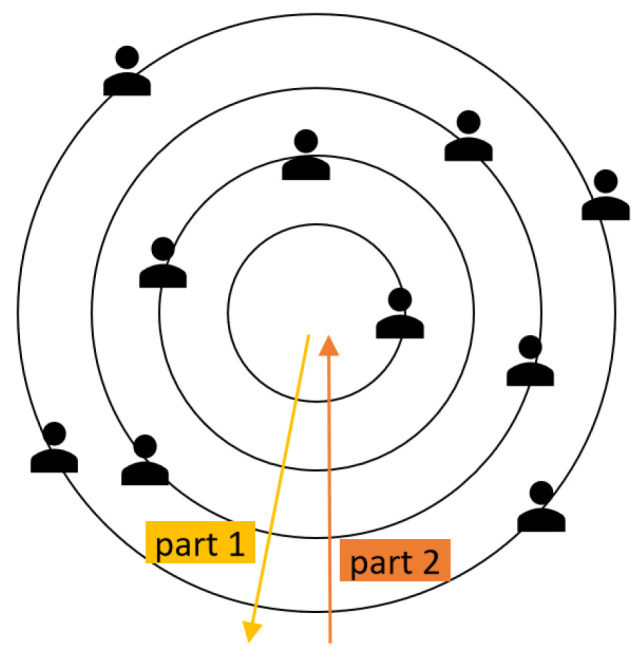
One negotiation round.

Once the formation of the groups has been determined, everything is ready for the negotiation process to begin. As shown in Figure 9, the groups, which are represented as agents in concentric circles, engage in the negotiation process, mimicking a pulsating rhythm. The negotiation process starts in the innermost circle, where the users of the lowest flexibility factors are grouped; then, their proposals are passed on to the second innermost circle, and this sequence continues outwards to the outermost circle. In the first part of the first round of negotiations, all circles pass on their preferred proposals to the outer circles, together with the proposals they have received from the inner circle or circles, regarding the position of the group circle and the number of groups. It is not effective to negotiate in the first part of the first round because, for example, the least flexible agents with very narrow views regarding deviations from the exact preference are very likely to fail to reach an agreement. Ideally, agents in the outer circles will easily adapt to the preferences of agents with lower flexibility factors due to their high flexibility, emphasizing a cooperative approach. However, it is important to avoid any form of exploitation and ensure that agents with higher flexibility factors are not forced into endless concessions.

After reaching the outermost circle, the first part of the negotiation round is over. The negotiation descends again towards the innermost circle, which is the second part of the negotiation round, as shown in Figure 9. When the process returns to the innermost circle, this marks a complete negotiation round, which can be compared to a heartbeat. In the second part of the first negotiation round, after reaching the outermost circle with the agents with the highest flexibility, the negotiation process only really begins when the agents analyze their circumstances and make a decision about their proposals.

To enable a more efficient and effective negotiation process, the agents’ proposals should be defined as a range around an agent’s exact preference and not just the exact preference itself. This approach, inspired by observations of human negotiations in which the use of ranges [51], albeit with a different focus, i.e., psychological, aimed to accelerate the negotiation process and reach constructive proposals. The range is logically defined around the exact preference, i.e., the anchor point.

In non-conflicting cases, the initial range proposals submitted by the agents overlap, facilitating a relatively straightforward negotiation process. However, in situations where these ranges do not overlap, a problematic situation arises. Ranges are achieved with the purpose of reaching a range of settings that facilitate potential agreement. For example, in a scenario with multiple agents, in which two or more agents’ ranges overlap but there is also a separate agent or multiple agents that do not have any overlaps, it is an obvious observation that the agent without overlapping ranges will be forced to adjust its proposed range to reach a mutually acceptable outcome. In scenarios where multiple agents are in this state, i.e., there is no overlap, the agent whose range is farthest from the other agents’ ranges is most likely to be pressured to adjust its proposals. This is because the described agent has the least likelihood of finding a mutually acceptable outcome with other agents with its current range, which makes it more susceptible to concessions, i.e., it is forced to expand its proposed range. If the described situation applies to more than one agent with the same distance to other agents’ ranges, the agent with the higher flexibility factor is expected to broaden its proposal range. In certain scenarios, an additional criterion comes into play—the size of the overlapping range. If two agents share an equal number of agents within their overlapping ranges, it is anticipated that the agent with the smaller overlapping range should be the one to expand its preferences. Once an agent deems an exact final proposal ready to be made, it is forwarded through all circles, i.e., all participants, for approval. If a consensus on the proposed setting is not achieved, the negotiation process continues.

As described, several criteria are taken into account when determining which agent will have to expand its range. These include the following: evaluating whether the agent’s proposed range overlaps with any other agent’s range, considering both the quantity of agent ranges it overlaps with, as well as the width of the overlapping; assessing the agent’s group based on its flexibility factor; analyzing the distance of the agent’s proposed range from other non-overlapping agents; and, in the case of ties, considering the number of concessions made in the current round. The pseudocode outlining the decision-making process for the user agents is presented in Algorithm 1, detailing the steps that the agents take upon receiving a query regarding their proposal. The computational complexity of the proposed algorithm is *O*(*n*), where *n* represents the number of user agents in the environment. The termination of the algorithm is guaranteed by its finite iterations and the bounded nature of the functions it calls. The completeness of the algorithm is obvious, and there are no segments that could cause incompleteness. For a more in-depth illustration of the factors and development of the negotiation process, detailed, step-by-step examples are provided in Section 4.

It may appear intuitive to assess the agreed-upon outcome and, with that, the entire negotiation process by relying on the comparison with the sum of the agents’ utility functions. However, this approach is not applicable in the observed use case, e.g., in a scenario in which two agents are present and have non-overlapping preference ranges. In such cases, optimizing the sum of their utility functions leads to the highest value if the chosen preferences exactly match the preference of one agent, resulting in a sum of 1. Unfortunately, the other agent is then completely dissatisfied (function at 0), which is an undesirable and unacceptable result.
**Algorithm 1** Agent logic to determine an answer to a proposition query  **Input:** Information about other agents (proposals, group organization)  **Output:** Proposal determined via the algorithm—unchanged or adapted range  1:**function** determineAnswer(peerInfo[])  2:                          ▹ Compare range overlapping  3:     lowestOverlapAgents[]←findLowestOverlap(peerInfo[])  4:     **if** lowestOverlapAgents[].containsOnly(thisAgent) **then**  5:        **return** newRangeCalculation()               ▹ Agent concedes  6:     **end if**  7:                             ▹ Compare flexibility rank  8:     higherFFactorAgent←thisAgent  9:     sameGroupAgents[]←[]10:   **for**  agentOther **in** lowestOverlapAgents[] **do**11:        **if** agentOther.group>thisAgent.group **then**12:           **return** currentProposal            ▹ Agent does not concede13:        **else if** agentOther.group=thisAgent.group **then**14:           sameGroupAgents.append(agentOther)15:        **end if**16:   **end for**17:   **if** higherFFactorAgent=thisAgent **and** sameGroupAgents.size()=0 **then**18:        **return** newRangeCalculation()             ▹ Agent concedes19:   **end if**20:                  ▹ Compare distance to next achievable overlap21:   mostDistanceAgent←thisAgent22:   tieAgents[]←[]23:   **for**  agentOther **in** sameGroupAgents[] **do**24:        distanceComparison←compareDistanceSum(thisAgent,agentOther)25:        **if** distanceComparison=1 **then**26:           mostDistanceAgent←agentOther27:           **return** currentProposal            ▹ Agent does not concede28:        **else if** distanceComparison=0 **then**29:           tieAgents.append(agentOther)30:        **end if**31:   **end for**32:   **if** len(tieAgents)=0 **and** mostDistanceAgent=thisAgent **then**33:        **return** newRangeCalculation()             ▹ Agent concedes34:   **end if**35:        ▹ Tie scenario (often in a 1 on 1 situation): compare concession count36:   **for** agentOther **in** tieAgents[] **do**37:        **if** thisAgent.roundConcessions>agentOther.roundConcessions **then**38:           **return** currentProposal            ▹ Agent does not concede39:        **end if**40:   **end for**41:   **return** currentProposal              ▹ Agent does not concede42:**end function**

## 4. Results and Discussion of the Negotiation Algorithm in the Smart Lighting Use Case

The results obtained with the described system were compared with our previous work, in which a centralized system approach was explored. In this study, a neural network was responsible for predicting the preferences of all users. The final decision on device settings was made by taking into account the number of users and their preferences using a calculation model. In this approach, users had no influence on the model’s decisions either through agents or other means. It is important to note that this approach does not take into account the flexibility factor, which was added later for comparison purposes. The comparison was based on the difference between the user’s exact preference and the selected device setting determined via the model in the centralized system, as opposed to the negotiation process in the multi-agent system. The results of the experiments conducted showed that the multi-agent system provided better results, with the overall deviation from the exact preferences, taking into account the flexibility factor, which was lower than in the centralized system with the calculation model.

In the remainder of this section, a comprehensive examination of the negotiation algorithm is presented using concrete cases. The showcased scenarios were carefully selected to illustrate the negotiation process in specific situations with a different number of agents and to highlight the differences in preferences and flexibility factors. To assess the algorithm’s efficacy, the segment involving the neural network’s prediction for each user via their agent was skipped”; i.e., exactly defined preferences were used to observe the negotiation process only, without the potential error of the neural network in preference prediction. In this analysis of the negotiation process using smart lighting as an example, the focus is on light intensity.

### 4.1. Experiment 1a

**Figure 10:**
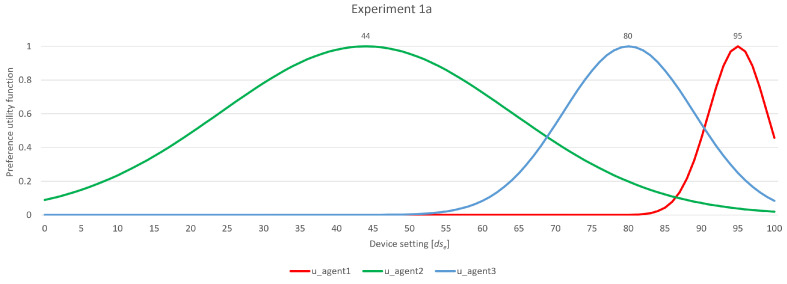
Experiment 1a—participating agents’ preferences and flexibility.

This experiment included agent profiles with the following preference and flexibility factor combinations (95, 4), (44, 20), and (80, 9), as depicted in Figure 10. In this example, according to their user preferences, two agents were in group 1, and one agent, with a flexibility factor of 20, was in group 2. The user agents initially defined their bargaining ranges, i.e., first propositions, as [91, 99] for *u_agent_1_*, [24, 64] for *u_agent_2_*, and [71, 89] for *u_agent_3_*, with no overlapping ranges observed.

A concise partial overview of the negotiation process is presented in Table 1. The table is organized by rounds, outlining the actions of each agent within each group across those rounds. Each row specifies which agent is presenting a proposition in a given group, accompanied by their “backed ranges”. The first value indicates the number of agents within their own group that share the same range, while the second value represents backing outside their group. This information is needed to determine which agent is required to broaden their range based on all agents’ proposals. In the last column of the table, the action of each agent is described, i.e., whether they estimate that they are not responsible for adjusting their proposed preference range to accommodate others or not. This type of table is used for the other examples in this paper as well.

Following the exchange of initial propositions in the first part of the first round (not shown in the table), in the second part, *u_agent_2_* in group 2 broadens its bargaining range from [24, 64] to [24, 74], as its initial range proposition did not overlap with any other. In this step, two negotiation occurrences come into view. Firstly, agents broaden their ranges only towards those of their counterparts, not in both directions. Secondly, the important decision of determining the extent by which the range expands must be made. Initially, all agents establish their ranges as [exact preference − σ, exact preference + σ]. As negotiations progress, the agents are compelled to expand their ranges sufficiently to either achieve an overlap or induce an opposing agent to expand its own range, i.e., removing that responsibility from itself. The governing principle specifies that the expansion required for this purpose is determined as the minimum between its σ value and the result of dividing σ by a natural number, denoted as n∈N. This strategic choice holds significance due to its adaptability to negotiation longevity. As negotiations progress into later rounds, the necessity for substantial expansions of the range gradually diminishes. This aligns seamlessly with agents’ intention to stay as close as possible to their initial exact preferences throughout the negotiation process.

With acquired backing now in their bargaining range, *u_agent_2_* forwards the proposition. In group 1, *u_agent_3_* determines overlapping with its range, which means it will stick to its proposition, while u_agent_1_ has to broaden its range due to a lack of backing. With this, *u_agent_1_* secures backing, i.e., overlapping with *u_agent_3_*. In this situation, it is clear that the absence of agreement is exclusive to *u_agent_1_* and *u_agent_2_*, while *u_agent_3_* aligns with both of them. Since it is determined that all agents in group 1 have backing, the amount of which is not individually lower than the amount of backing for agents in other groups, the negotiation process enters round 2, reaching group 2 again. Requesting *u_agent_2_* to align solely with *u_agent_1_*, given its high flexibility factor, would be unfair. However, as the negotiation process approaches its finalization, it is imperative to consider the difference in flexibility factors. Taking into account the frequency of concessions made by each agent is another factor under consideration. Given this, *u_agent_3_* is ready to make a proposal to prevent a continual back-and-forth widening of ranges between *u_agent_1_* and *u_agent_2_*. In this instance, the proposal is accepted by all agents. The same principle would apply if only *u_agent_1_* and *u_agent_2_* were present. It would be unjust to require *u_agent_2_* to cover the entire distance between its ranges solely due to its higher flexibility factor. An agreement is reached regarding the agents’ flexibility factors, but this does not imply that the responsibility lies solely with the agent possessing higher flexibility. Both agents are mandated to expand their ranges accordingly.

### 4.2. Experiment 1b

**Figure 11:**
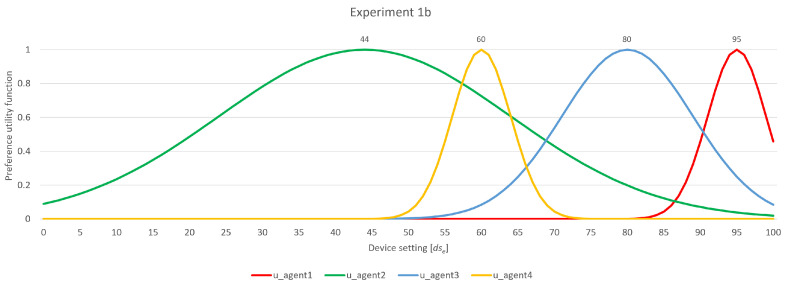
Experiment 1b—participating agents’ preferences and flexibility.

This experiment built on the previous example by adding another agent, *u_agent_4_*, with the preference and flexibility factor combination of (60, 4), as shown in Figure 11. Following the low flexibility factor of *u_agent_4_*, it is added to group 1, which means group 2 is unchanged; i.e., only *u_agent_2_* remains in group 2. Right at the beginning of the negotiation process, in comparison to Experiment 1a, there is a difference visible in Table 2. In the previous experiment, *u_agent_2_*, the most flexible agent, was forced to broaden its range at the beginning because it had no overlap. Since that is not the case now, because of the overlap with the range of *u_agent_4_*, *u_agent_2_* now forwards its proposal range to group 1. In group 1, *u_agent_4_* is the only one with overlapping, leaving agents *u_agent_1_* and *u_agent_3_* as candidates to broaden their ranges. As *u_agent_1_* is furthest from other agents’ ranges, it is the one to broaden its range. After widening, *u_agent_1_*’s gain is not exclusive; *u_agent_3_* now benefits from an overlap supported by *u_agent_1_*’s expansion.

At this point, all user agents of group 1 determine that they have the same backing quantity among all agents, and, as there is a more outer circle, they forward their current ranges to the more outer circle that houses agents with a higher flexibility factor. Now, *u_agent_2_* is forced to broaden its range. Following this, it now overlaps with *u_agent_3_* and forwards its proposition back to group 1. Here, *u_agent_3_* has backing with two other agents, so the decision is up to *u_agent_1_* or *u_agent_4_*, which both have the same backing quantity; i.e., they are both backed by one other agent. The decision to broaden its range is made by *u_agent_1_*, as its backing range is smaller than that of *u_agent_4_*. With the introduction of the newly proposed range, *u_agent_1_* is now backed by two agents, whereas *u_agent_4_* remains backed by only one. As a result, *u_agent_4_* needs to expand its range, allowing for a final proposal of 72, which, in this case, is agreed upon by all agents.

### 4.3. Experiment 2

**Figure 12:**
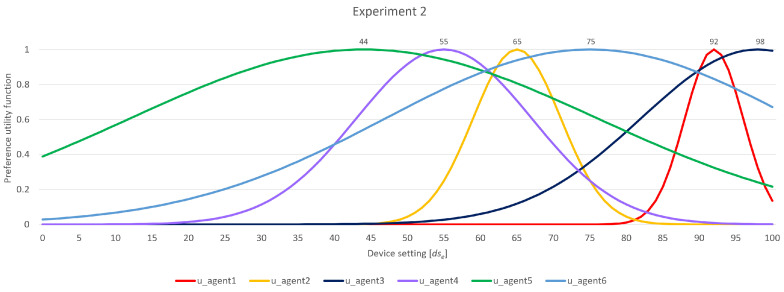
Experiment 2—participating agents’ preferences and flexibility.

In this experiment, a scenario was examined involving three groups, each composed of two agents, totaling six agents. The most flexible agents, *u_agent_5_* and *u_agent_6_*, were in group 3. Group 2 consisted of *u_agent_3_* and *u_agent_4_*, representing moderate flexibility in this scenario. Meanwhile, group 1 included the least flexible agents, *u_agent_1_* and *u_agent_2_*. The exact preferences and width of agents’ flexibility functions are shown in Figure 12.

This example illustrates a scenario in which the most flexible agents were not making substantial changes to their ranges since they already encompassed a significant portion of other agents’ ranges. Following the initial phase of the first round, the agents in group 3 assess their backings. Agent *u_agent_6_* exhibits an overlap with all other agents (one within the same group and four in other groups), while *u_agent_5_* has an overlap with three of the five agents in total. After their analysis, they decide that no adjustments to their ranges are needed presently to reach an agreement. They proceed to pass on their current proposed ranges to group 2.

Agent *u_agent_3_* of group 2 identifies the necessity to expand its range. This decision is influenced by having the least support and being situated in a circle further out. Even though *u_agent_1_* in group 1 has the same quantity of backing as other agents, *u_agent_3_* broadens its range due to higher flexibility. Now, *u_agent_3_* (and *u_agent_4_*) is in a position to forward its proposition since there are agents that have to broaden their ranges based on the set principles. In group 1, agent *u_agent_1_* overlaps with only two agents in terms of ranges; it is the lowest among all agents in the current negotiation process. Consequently, there is a need for *u_agent_1_* to expand its bargaining range. As a result, both the agents in group 1 overlap with the ranges of three other agents. Although this is the lowest quantity, the agents in outer circles also share the same quantity. Consequently, they move forward to submit their proposals to group 2, as there are more eligible agents that should expand their bargaining ranges.

Both agents in group 2 have overlaps with the ranges of three other agents. *u_agent_4_* is the one to broaden its range, given that its range is the farthest from the ranges of the other agents. This is another criterion taken into account when making decisions. After *u_agent_4_*’s move, *u_agent_3_* now acknowledges the necessity of broadening its range since it is the agent with the range furthest away from the other agents’ ranges and the least overlap with backed ranges. With this broadening, *u_agent_3_* achieves an overlap with *u_agent_4_*, providing both agents in group 2 with enough backing to advance their proposals. Given that all agents in group 3 already cover the ranges of every other agent, there is no pressure for them to broaden their ranges. As a result, the negotiation process returns to group 1.

Now, with all other agents outside of group 1 achieving an overlap of their bargaining ranges, the responsibility falls on *u_agent_1_* and *u_agent_2_* despite their being the less flexible agents. In this context, as *u_agent_1_* is the farthest from everyone else, it is the one to broaden its range. Taking the opportunity, *u_agent_1_* puts forward a final proposal that leans more toward its initial preference and bargaining range. Ultimately, this proposal is accepted by *u_agent_2_*, as it has not made any changes to its ranges until now, and the proposed range is not significantly distant from its preferred range. All the other agents also agree, as the proposal falls within their bargaining ranges. The summarized overview of the negotiation process is given in Table 3.

## 5. Conclusions

This paper has presented a context-aware, multi-agent system designed to improve service provisioning in smart spaces. It enables the simultaneous consideration of the different preferences of multiple users via negotiations among user agents without user intervention. The proposed system is a viable solution to the gap in the provision of ambient services caused by the lack of solutions for multiple users at the same time, i.e., when users are affected by the same device settings when they are in the same environment at the same time. First, the current state of ambient service provisioning was analyzed, considering different approaches that incorporate intelligent services in smart spaces. Then, the multi-agent system was presented in detail with a description of the different agents and their tasks and obligations, as well as a context-dependent preference definition and the prediction of preferences for a previously unseen context using an ANN. The main contribution is the negotiation algorithm and the organization of negotiation participants, which support multiple users and incorporate all user preferences into the negotiation process without neglecting any of them. In addition, the system incorporates user flexibility factors that users determine and use to express their willingness and openness to settings that differ from their preferences. The negotiation process was illustrated using the smart lighting use case, offering a comprehensive description of the entire system within this selected application.

The results of the selected scenarios have been presented, followed by a discussion of the proposed negotiation algorithm and its organization. As has been mentioned in the paper, many researchers point out the problem of user satisfaction when there are multiple users with different preferences. The presented system offers a new way to effectively provide services to multiple users without user intervention. It attempts to satisfy all users by employing software agents that negotiate and advocate for the preferences of their users during agent negotiations. It is planned to further develop this system in order to achieve the best-quality user experience with the shortest possible negotiation, i.e., with as little need for message transactions in the negotiation process as possible. The ongoing challenge is to further optimize the responsiveness of the system to context changes so that they appear to go unnoticed by users with a focus on user changes, i.e., when a user enters or leaves the smart space. Another planned optimization in future work is to manage more than one service simultaneously with the same multi-agent system. The aim is to improve the ability of the multi-agent system to handle multiple services simultaneously. This goes beyond simultaneous negotiations; it includes upgrading the negotiation system to manage interdependent negotiation processes. For example, consider negotiations concerning light color and light intensity.

## Figures and Tables

**Table 1:** Experiment 1a—rough partial overview of the negotiation process.

Round 1—Group 2			
textitu_agent_2_	proposition: [24, 64]	backed ranges: [0/2][0/0]	→ broaden range [24, 74]
*u_agent_2_*	proposition: [24, 74]	backed ranges: [1/2][0/0]	→ forward proposition
Round 1/2—Group 1			
*u_agent_3_*	proposition: [71, 89]	backed ranges: [0/1][1/1]	→ forward proposition
*u_agent_1_*	proposition: [91, 99]	backed ranges: [0/1][0/1]	→ broaden range [89, 99]
*u_agent_3_*	proposition: [71, 89]	backed ranges: [1/1][1/1]	→ forward proposition
*u_agent_1_*	proposition: [89, 99]	backed ranges: [1/1][0/1]	→ forward proposition
Round 2—Group 2			
*u_agent_2_*	proposition: [24, 74]	backed ranges: [0/0][1/2]	→ broaden range
			→ final proposal 87

**Table 2:** Experiment 1b—rough partial overview of the negotiation process.

Round 1—Group 2			
*u_agent_2_*	proposition: [24, 64]	backed ranges: [0/0][1/3]	→ forward proposition
Round 1/2—Group 1			
*u_agent_3_*	proposition: [71, 89]	backed ranges: [0/2][0/1]	→ forward proposition
*u_agent_1_*	proposition: [91, 99]	backed ranges: [0/2][0/1]	→ broaden range [89, 99]
*u_agent_4_*	proposition: [56, 64]	backed ranges: [0/2][1/1]	→ forward proposition
*u_agent_3_*	proposition: [71, 89]	backed ranges: [1/2][0/1]	→ forward proposition
*u_agent_1_*	proposition: [89, 99]	backed ranges: [1/2][0/1]	→ forward proposition
*u_agent_4_*	proposition: [56, 64]	backed ranges: [0/2][1/1]	→ forward proposition
Round 2—Group 2			
*u_agent_2_*	proposition: [24, 64]	backed ranges: [0/0][1/3]	→ broaden range [24, 74]
*u_agent_2_*	proposition: [24, 74]	backed ranges: [0/0][2/3]	→ forward proposition
Round 2—Group 1			
*u_agent_3_*	proposition: [71, 89]	backed ranges: [1/2][1/1]	→ forward proposition
*u_agent_1_*	proposition: [89, 99]	backed ranges: [1/2][0/1]	→ broaden range [81, 99]
*u_agent_4_*	proposition: [56, 64]	backed ranges: [0/2][1/1]	→ forward proposition
*u_agent_3_*	proposition: [71, 89]	backed ranges: [1/2][1/1]	→ forward proposition
*u_agent_1_*	proposition: [81, 99]	backed ranges: [1/2][1/1]	→ forward proposition
*u_agent_4_*	proposition: [56, 72]	backed ranges: [1/2][1/1]	→ broaden range
			→ final proposal 72

**Table 3:** Experiment 2—rough partial overview of the negotiation process.

Round 1—Group 3			
*u_agent_5_*	proposition: [12, 76]	backed ranges: [1/1][2/4]	→ forward proposition
*u_agent_6_*	proposition: [43, 100]	backed ranges: [1/1][4/4]	→ forward proposition
Round 1—Group 2			
*u_agent_3_*	proposition: [82, 100]	backed ranges: [0/1][2/4]	→ broaden range [74, 100]
*u_agent_4_*	proposition: [43, 67]	backed ranges: [0/1][3/4]	→ forward proposition
*u_agent_3_*	proposition: [74, 100]	backed ranges: [0/1][3/4]	→ forward proposition
*u_agent_4_*	proposition: [43, 67]	backed ranges: [0/1][3/4]	→ forward proposition
Round 1/2—Group 1			
*u_agent_1_*	proposition: [88, 96]	backed ranges: [0/1][2/4]	→ broaden range [76, 96]
*u_agent_2_*	proposition: [59, 71]	backed ranges: [0/1][3/4]	→ forward proposition
*u_agent_1_*	proposition: [76, 96]	backed ranges: [0/1][3/4]	→ forward proposition
*u_agent_2_*	proposition: [59, 71]	backed ranges: [0/1][3/4]	→ forward proposition
Round 2—Group 2			
*u_agent_3_*	proposition: [74, 100]	backed ranges: [0/1][3/4]	→ forward proposition
*u_agent_4_*	proposition: [43, 67]	backed ranges: [0/1][3/4]	→ broaden range [43, 73]
*u_agent_3_*	proposition: [74, 100]	backed ranges: [0/1][3/4]	→ broaden range [72, 100]
*u_agent_4_*	proposition: [43, 73]	backed ranges: [0/1][3/4]	→ forward proposition
*u_agent_3_*	proposition: [72, 100]	backed ranges: [1/1][3/4]	→ forward proposition
*u_agent_4_*	proposition: [43, 73]	backed ranges: [1/1][3/4]	→ forward proposition
Round 2/3—Group 1			
*u_agent_1_*	proposition: [76, 96]	backed ranges: [0/1][3/4]	→ broaden range
			→ final proposal 73
*u_agent_2_*	proposition: [59, 71]	backed ranges: [0/1][4/4]	→ forward proposition

## Data Availability

The original contributions presented in the study are included in the article.

## Abbreviations

The following abbreviations are used in this manuscript:

AAL	Ambient assisted living
AAN	Artificial neural network
AI	Artificial intelligence
AmI	Ambient intelligence
CIoT	Cognitive Internet of Things
IoT	Internet of Things
MAS	Multi-agent system
SC	Silhouette coefficient
